# A large survey among European trainees in clinical microbiology and infectious disease on training systems and training adequacy: identifying the gaps and suggesting improvements

**DOI:** 10.1007/s10096-016-2791-9

**Published:** 2016-10-04

**Authors:** E. Yusuf, D. S. Y. Ong, A. Martin-Quiros, C. Skevaki, J. Cortez, K. Dedić, A. E. Maraolo, D. Dušek, P. J. Maver, M. Sanguinetti, E. Tacconelli, The Trainee Association of the European Society of Clinical Microbiology and Infectious Diseases (ESCMID)

**Affiliations:** 1 Department of Medical Microbiology and Infection Prevention, Universitair Ziekenhuis Brussel, Brussels, Belgium; 2 Department of Medical Microbiology, Universitair Ziekenhuis Antwerpen, University of Antwerp, Edegem, Belgium; 3 Department of Medical Microbiology, University Medical Center Utrecht, Utrecht, The Netherlands; 4 Emergency Department, Hospital Universitario La Paz—Instituto de Investigación del Hospital Universitario La Paz, Madrid, Spain; 5 University Hospital Giessen and Marburg GmbH, Philipps University, Marburg, Germany; 6 Infectious Diseases Department, Centro Hospitalar e Universitário de Coimbra, Coimbra, Portugal; 7 CISA, Health Research Centre of Angola, Caxito, Angola; 8 Microbiology Department, Cantonal Hospital “Dr. Irfan Ljubijankic”, Bihac, Bosnia and Herzegovina; 9 Department of Clinical Medicine and Surgery, Section of Infectious Diseases, University of Naples “Federico II”, Naples, Italy; 10 University Hospital for Infectious Diseases “Dr. Fran Mihaljevic”, Zagreb, Croatia; 11 Institute of Microbiology and Immunology, Faculty of Medicine, University of Ljubljana, Ljubljana, Slovenia; 12 l’Istituto di Microbiologia, Universita Cattolica del Sacro Cuore, Rome, Italy; 13 Infectious Diseases, University Hospital Tübingen, DZIF Center, Tübingen, Germany

## Abstract

The purpose of this investigation was to perform a survey among European clinical microbiology (CM) and infectious disease (ID) trainees on training satisfaction, training tools, and competency assessment. An online, anonymous survey in the English language was carried out between April and July 2015. There were 25 questions: seven in a 5-point Likert scale (1: worst scenario, 5: best scenario) and the remainder as closed multiple-choice questions in five areas (satisfaction, adequacy, system, mentorship, and evaluation of training). Included were 419 respondents (215 CM, 159 ID, and 45 combined CM/ID) from 31 European countries [mean age (standard deviation) 32.4 (5.3) years, 65.9 % women]. Regarding satisfaction on the training scheme, CM and ID scored 3.6 (0.9) and 3.2 (1.0), respectively. These scores varied between countries, ranging from 2.5 (1.0) for Italian ID to 4.3 (0.8) for Danish CM trainees. The majority of respondents considered training in management and health economics inadequate and e-learning and continuing medical education programs insufficient. Many trainees (65.3 % of CM and 62.9 % of ID) would like to have more opportunities to spend a part of their training abroad and expected their mentor to be more involved in helping with future career plans (63.5 % of CM and 53.4 % of ID) and practical skills (53.0 % of CM and 61.2 % of ID). Two-thirds of the respondents across the specialties agreed that a European exam should be developed, but half of them thought it should not be made mandatory. This survey shows high heterogeneity in training conditions in European countries, identifies perceived gaps in training, and suggests areas for improvements.

**Electronic supplementary material:**

The online version of this article (doi:10.1007/s10096-016-2791-9) contains supplementary material, which is available to authorized users.

## Introduction

Clinical microbiology (CM) and infectious disease (ID) are two closely related medical specialties that deal with the diagnosis, management, and control of infectious disease [1, 2]. Unlike many other medical specialties in Europe, CM and ID are not uniformly recognized as a distinct medical profession, and the training requirements vary between European countries [2]. This variation might limit cross-border training and could be counterproductive in treating infections, since pathogens do not respect national borders [3, 4]. These specialties are also facing specific challenges, such as increasing patient mobility and immigration, and major technological advances, including rapid diagnostics and point-of-care tests [5]. CM and ID might also overlap, for example due to changes in CM professional tasks in several countries. The profession may evolve from being mainly laboratory-based to a more clinical profile including diagnostic testing, therapeutics, and infection control [1, 6, 7].

Several authors have recognized the need to respond to these new challenges and proclaim that the training of CM and ID specialists should be improved [1, 2, 6], although no survey including European trainees has been performed yet.

The aim of the present study was to assess the European CM and ID trainees’ training satisfaction rate, the adequacy of the training parts and tools, and the monitoring of competency.

## Materials and methods

### Survey strategy

Survey questions were developed by the members of the Trainee Association of the European Society of Clinical Microbiology and Infectious Diseases (TAE ESCMID) steering committee. Among the goals of the TAE are trying to shape an optimal training program in CM and ID, and increasing collaboration between European trainees. The survey was first tested among 32 CM and ID trainees from six European countries to review whether the questions were understandable. After necessary amendments, the survey was launched on April 25th, 2015 during the European Clinical Microbiology and Infectious Disease (ECCMID) congress in Copenhagen, Denmark. During the ECCMID, the survey was advertised on the screens in the congress venue and announced during the trainees’ day session. Trainees were also actively approached in person to fill in the survey. After the ECCMID, the survey ran online on the open source software Lime Survey (LimeSurvey GmbH, Hamburg, Germany) until July 13th, 2015, during which time trainees were approached by social media, the ESCMID website, and by local TAE representatives in almost all countries in Europe.

Trainees or young specialists (within 3 years after finishing the training) in CM and ID from European countries were eligible for this survey. The survey was essentially anonymous. In the survey, respondents were first asked to fill in demographic characteristics, type of residency training (CM, ID, or combined CM/ID), primary work places, salaries after tax, and accreditation status of their training (eight questions in total). The present survey further covered five areas using 25 questions. A 5-point Likert scale (1: worst scenario, 5: best scenario) was applied for seven questions and the remainder were closed multiple-choice questions. The first part covered the rate of satisfaction with their training scheme and curriculum, and reason for dissatisfactions, if any. The second part focused on training adequacy in the following areas: informatics, health economics, travel medicine, management (administration), infection control, and transplantation medicine. The third part questioned training methods: theory, seminars, practical exercise, e-learning, and the opportunity to spend time abroad. The participants were also asked about their weekly/regular program: didactic session by attendees/residents, quizzes, study groups, and journal clubs and availability of online journal access. The fourth part focused on the availability of a mentorship program as well as (extended) needs for this. The fifth part of the survey evaluated training assessment, including type and frequency. The survey also checked methods for progress evaluation: logbook, quizzes, continuing medical education (CME) program, direct observation, and formal examination at the end of the training. Lastly, the survey participants were asked whether they would favor a European exam for ID and/or CM. It was estimated that 15 min was needed to answer all questions. The questionnaire of the survey is available as Supplementary File 1.

### Statistical analysis

The results were collated by proMENTE Social Research (Sarajevo, Bosnia and Herzegovina). Categorical variables were summarized with frequencies and percentages, and continuous variables were summarized by means and standard deviation (SD). The responses to the 5-point Likert scale were considered to be equidistant and symmetric, and summarized as mean (SD). The results were analyzed separately for CM and ID, since the daily activities of these two specializations are different, and whenever possible (due to the low number of respondents) for CM/ID. To show regional differences in the rate of satisfaction with their training scheme and curriculum and salaries, the countries of participants were categorized into European regions as used by United Nations Statistics Division [8]: Eastern (Belarus, Bulgaria, Czech Republic, Hungary, Poland, Romania), Northern (Denmark, Estonia, Finland, Ireland, Latvia, Lithuania, Norway, Sweden, United Kingdom), Southern (Bosnia and Herzegovina, Croatia, Greece, Italy, Portugal, Slovenia, Spain), Western (Austria, Belgium, France, Germany, The Netherlands, Switzerland), and other European countries (Azerbaijan and Turkey). The differences in the regions concerned were investigated using one-way analysis of variance (ANOVA). No further statistical analyses were performed because this survey did not test any specific hypothesis. All analyses were performed using IBM SPSS Statistics for Windows version 23.0 (IBM Corp., Armonk, NY).

## Results

### Demographic characteristics of the respondents

There were 419 respondents [mean age (SD) 32.4 (5.3) years, 65.9 % women] who completed the survey (Table 1). The respondents represented 31 European countries: 23 members of the European Union (EU), two members of the European Economic Area (EEA)/Switzerland, and six other European countries (Table 1). Pooled in regions, the most respondents came from Southern Europe (47.7 %), followed by Western (28.6 %), Northern (10.0 %), Other (9.5 %), and Eastern Europe (4.1 %). The number of respondents varied from one (Estonia, Poland, Kosovo, and Macedonia) to 54 (Italy) per country. Eleven countries (Austria, Belgium, Croatia, Denmark, France, Germany, Italy, The Netherlands, Portugal, Slovenia, and Spain) had ≥5 CM respondents and seven countries (Croatia, France, Italy, Portugal, Spain, Switzerland, and Norway) had ≥5 ID respondents. There was a significant difference between regions regarding salaries, mean (SD) from high to low in Euro: Northern [4525 (2400)], Western [3629 (1815)], Other Europe [1727 (697)], Southern [1470 (610)], and Eastern [590 (239)].

**Table 1:** Characteristics of the respondents

Countries	Number of trainees stratified by medical specialty			% women	Mean monthly salaries after tax in Euro (SD)	Primary work place, %			Accredited scheme, %		
	CM	ID	CM/ID			University	Large non-university	Small or private clinic	No	Yes	No idea
Overall	215	159	45	66	2375 (1789)	76.6	13.1	10.3	13.4	52.8	33.8
European Union countries											
Austria (*n* = 12)	8	2	2	54	2755 (954)	75	16.7	8.3	9.1	36.4	54.5
Belgium (*n* = 13)	11	1	1	50	2366 (563)	53.8	38.5	7.7	7.7	30.8	61.5
Bulgaria (*n* = 2)	0	2	0	0	600 (141)	100	0	0	0	50	50
Croatia (*n* = 40)	19	19	2	17	1295 (398)	72.5	7.5	20	23.1	25.6	51.3
Czech Republic (*n* = 2)	1	1	0	0	370 (0)	100	0	0	50.0	50.0	0
Denmark (*n* = 2)	6	1	0	75	4394 (1966)	85.7	14.3	0	0	28.6	71.4
Estonia (*n* = 1)	1	0	0	0	1000	100	0	0	0	100	0
France (*n* = 34)	17	15	2	53	3083 (1273)	73.5	20.6	5.9	12.1	54.5	33.3
Germany (*n* = 13)	9	4	0	46	3367 (1202)	92.3	7.7		16.7	58.3	25.0
Greece (*n* = 10)	4	4	2	80	1356 (634)	40	40	20	12.5	25	62.5
Hungary (*n* = 7)	3	4	0	71	753 (152)	57.1	14.3	28.6	0	71.4	28.6
Ireland (*n* = 3)	2	1	0	67	4900 (565)	100	0	0	0	66.7	33.3
Italy (*n* = 53)	16	27	10	74	1362 (810)	86.8	7.5	5.7	1.9	61.2	36.7
Lithuania (*n* = 5)	3	2	0	60	487 (172)	100	0	0	0	100	0
Malta (*n* = 2)	1	1	0	50	1800 (0)	100	0	0	50	0	50
The Netherlands (*n* = 35)	33	1	1	67	3687 (1442)	85.7	5.7	8.6	0	84.8	15.2
Poland (*n* = 1)	1	0		0	650	100	0	0	0	100	0
Portugal (*n* = 38)	8	28	2	71	1627 (476)	60.5	15.8	23.7	8.1	62.2	29.7
Romania (*n* = 2)	2	0	0	0	600 (283)	100	0	0	0	50	50
Slovenia (*n* = 12)	7	5	0	83	1683 (338)	100	0	0	0	33.3	66.7
Spain (*n* = 38)	28	8	2	66	1698 (544)	78.9	2.6	18.4	17.6	73.5	8.8
Sweden (*n* = 4)	2	1	1	75	4210 (1124)	100	0	0	0	33.3	66.7
United Kingdom (*n* = 8)	3	1	4	13	5362 (2117)	75	25	0	0	87.5	12.5
European Economic Area countries and Switzerland											
Switzerland (*n* = 13)	0	12	1	46	7015 (1685)	92.9	7.1	0	0	100	0
Norway (*n* = 14)	2	11	1	57	5819 (1826)	71.4	28.6	0	21.4	42.9	35.7
Others											
Azerbaijan (*n* = 1)	1	0	0	50	127 (0)	50	50	0	50	0	50
Belarus (*n* = 3)	0	2	1	67	247 (119)	66.7	33.3	0	0	66.7	33.3
Bosnia and Herzegovina (*n* = 5)	4	0	1	80	955 (270)	33.3	66.7	0	50	0	50
Kosovo (*n* = 1)	0	1	0	0	530	No data	No data	No data	0	100	0
Macedonia (*n* = 1)	1	0	0	0	450	No data	No data	No data	0	100	0
Turkey (*n* = 39)	22	5	12	69	1769 (649)	74.4	12.8	12.8	50.0	11.8	38.2

### Training satisfaction

**Fig. 1:**
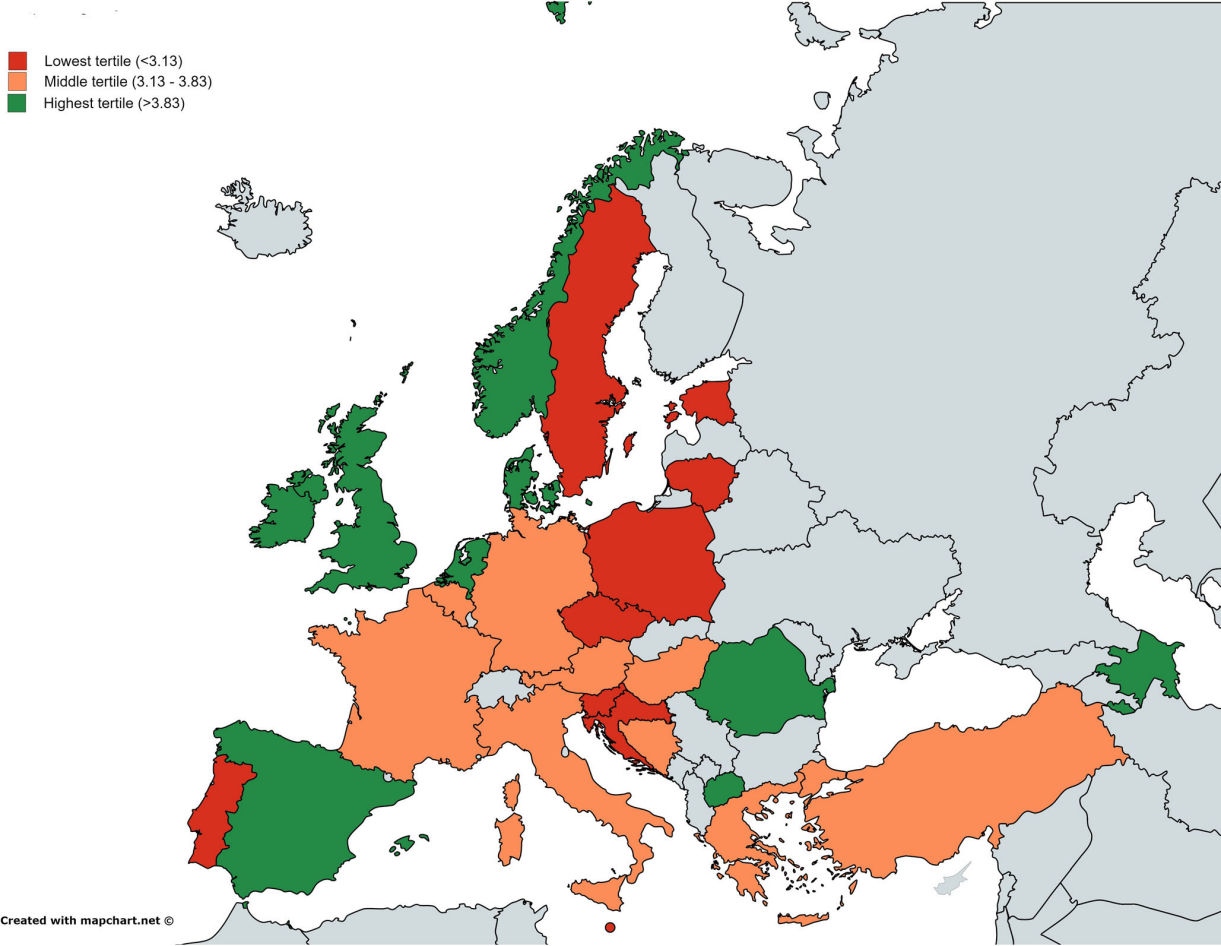
Map of the mean satisfaction score of clinical microbiology trainees regarding their training in a given European country. The three colors (green, orange, and red) represent the mean score in tertiles without weighting for the number of participants of each country

**Fig. 2:**
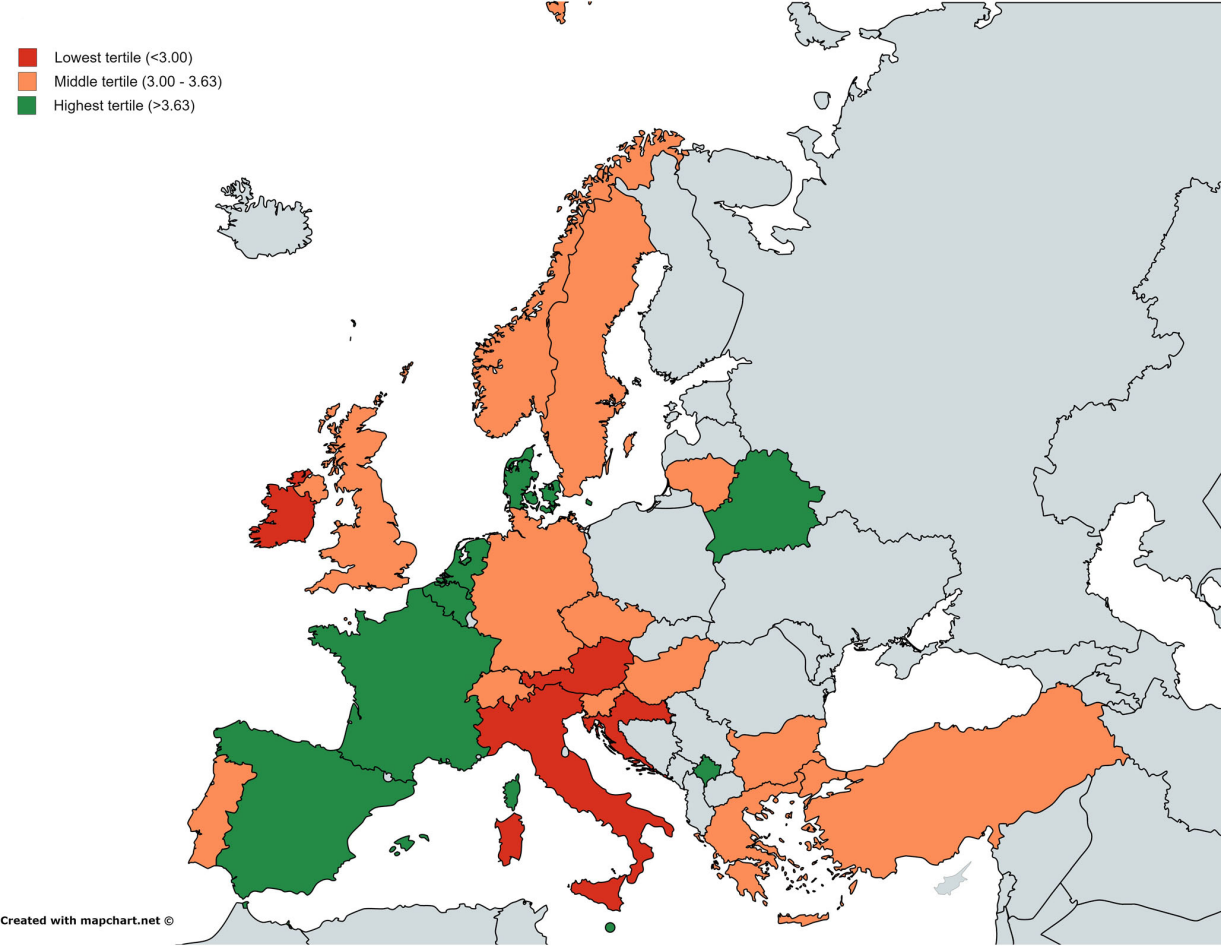
Map of the mean satisfaction score of infectious disease trainees regarding their training in a given European country. The three colors (green, orange, and red) represent the mean score in tertiles without weighting for the number of participants of each country

CM trainees reported a trend of being satisfied with their training scheme [mean (SD) of 3.6 (0.9), Table 2 and Fig. 1]. Among countries with ≥5 CM respondents, CM trainees from Denmark were the most satisfied with their training program [4.3 (0.8)] and Slovenian CM trainees were the least satisfied [2.7 (0.8)]. ID trainees reported a mean satisfaction score of 3.2 (1.0) (Table 3 and (Fig. 2). French ID trainees were the most satisfied [3.9 (0.9)] and Italian ID trainees were the least [2.5 (1.0)]..

**Table 2:** Clinical microbiology trainees’ rating on satisfaction of their training and on training adequacy

Countries	Satisfaction of training scheme (1: completely dissatisfied, 5: completely satisfied), mean (SD)	Training adequacy (1: completely inadequate, 5: completely adequate), mean (SD)					
		Informatics	Health economics	Travel medicine	Management	Infection control	Transplantation medicine
Overall (*n* = 215)	3.6 (0.9)	3.2 (1.0)	2.6 (1.0)	3.0 (1.0)	2.9 (0.9)	3.7 (0.7)	2.6 (1.0)
European Union countries							
Austria (*n* = 8)	3.7 (0.8)	3.7 (0.5)	3.5 (0.5)	3.2 (0.8)	3.3 (0.5)	4.0 (0)	2.5 (0.5)
Belgium (*n* = 11)	3.7 (0.5)	3.7 (0.8)	2.3 (1.1)	2.7 (0.7)	3.3 (0.7)	3.7 (0.8)	2.9 (0.8)
Bulgaria (*n* = 0)	–	–	–	–	–	–	–
Croatia (*n* = 19)	3.1 (1.0)	2.9 (0.8)	2.5 (0.9)	2.8 (1.1)	2.7 (1.0)	3.3 (1.0)	3.5 (0.7)
Czech Republic (*n* = 1)	3.0	3.0	3.0	2.0	3.0	3.0	3.0
Denmark (*n* = 6)	4.3 (0.8)	3.5 (1.0)	3.0 (1.3)	3.5 (0.8)	3.3 (0.8)	4.0 (0.6)	2.7 (1.2)
Estonia (*n* = 1)	3.0	4.0	2.0	2.0	2.0	3.0	3.0
France (*n* = 17)	3.6 (0.8)	3.0 (1.1)	2.8 (0.8)	3.3 (0.8)	2.8 (0.7)	3.9 (0.3)	2.7 (0.8)
Germany (*n* = 9)	3.5 (0.8)	3.9 (0.3)	3.4 (0.9)	3.1 (0.6)	2.9 (1.0)	3.6 (0.5)	2.9 (0.6)
Greece (*n* = 4)	3.3 (0.6)	4.0	3.0	3.0	4.0	3.0	2.0
Hungary (*n* = 3)	3.3 (0.6)	2.7 (1.5)	1.7 (0.6)	1.7 (0.6)	2.7 (0.6)	2.7 (1.2)	3.3 (1.2)
Ireland (*n* = 2)	4.0 (0)	3.0 (0)	3.0 (0)	2.0 (0)	3.0 (0)	3.5 (0.7)	2.0
Italy (*n* = 16)	3.2 (1.1)	2.7 (1.0)	2.1 (0.9)	2.4 (1.0)	2.3 (1.1)	3.3 (0.8)	3.2 (1.2)
Lithuania (*n* = 3)	2.7 (1.2)	2.0 (1.7)	2.0 (1.0)	3.3 (0.6)	2.7 (1.5)	4.0 (0)	2.3 (1.2)
Malta (*n* = 1)	3.0	3.0	2.0	2.0	3.0	4.0	2.0
The Netherlands (*n* = 33)	4.0 (0.8)	3.6 (0.9)	2.7 (0.8)	3.4 (0.8)	3.2 (0.9)	4.2 (0.4)	1.8 (0.5)
Poland (*n* = 1)	3.0	3.0	3.0	3.0	3.0	4.0	2.0
Portugal (*n* = 8)	3.0 (1.0)	3.6 (0.5)	2.6 (0.8)	2.8 (1.2)	2.2 (1.0)	3.1 (0.7)	3.0 (0.9)
Romania (*n* = 2)	4.5 (0.7)	3.0 (1.4)	2.5 (0.7)	3.0 (0)	3.5 (0.7)	4.1 (0.4)	2.5 (2.1)
Slovenia (*n* = 7)	2.7 (0.8)	2.7 (1.1)	1.7 (0.8)	3.1 (1.2)	2.6 (1.0)	4.0 (0.6)	2.1 (0.4)
Spain (*n* = 28)	3.9 (0.7)	3.4 (1.0)	2.8 (1.1)	3.4 (1.0)	3.0 (1.0)	3.0 (1.0)	2.2 (0.7)
Sweden (*n* = 4)	3.0	2.0	4.0	1.0	3.0	1.0	4.0
United Kingdom (*n* = 3)	4.3 (0.6)	3.3 (1.5)	3.0 (1.7)	3.5 (0.7)	3.0 (1.7)	3.0 (1.4)	1.3 (0.6)
European Economic Area countries and Switzerland							
Switzerland (*n* = 0)	–	–	–	–	–	–	–
Norway (*n* = 2)	4.5 (0.7)	4.0 (0)	4.0 (0)	4.5 (0.7)	4.0 (0)	5.0 (0)	1.5 (0.7)
Others							
Azerbaijan (*n* = 1)	4.0	4.0	2.0	2.0	4.0	1.0	5.0
Belarus (*n* = 0)	–	–	–	–	–	–	–
Bosnia and Herzegovina (*n* = 4)	3.3 (1.5)	2.7 (1.2)	2.0 (1.7)	1.3 (0.6)	2.7 (1.1)	3.3 (0.6)	4.0 (0.8)
Kosovo (*n* = 0)	–	–	–	–	–	–	–
Macedonia (*n* = 1)	5.0	No data	No data	No data	3.0	4.0	No data
Turkey (*n* = 22)	3.2 (1.0)	3.1 (0.7)	2.4 (0.9)	2.6 (0.7)	3.0 (0.7)	3.4 (0.7)	3.3 (0.9)

**Table 3:** Infectious disease trainees’ rating on satisfaction of their training and on training adequacy

Countries	Satisfaction of training scheme (1: completely dissatisfied, 5: completely satisfied), mean (SD)	Training adequacy (1: completely inadequate, 5: completely adequate), mean (SD)					
		Informatics	Health economics	Travel medicine	Management	Infection control	Transplantation medicine
Overall (*n* = 159)	3.2 (1.0)	2.9 (1.0)	2.4 (0.9)	3.1 (1.1)	2.4 (0.9)	3.3 (1.0)	3.1 (1.2)
European Union countries							
Austria (*n* = 2)	2.5 (0.7)	3.0	2.0 (0)	3.0 (0)	2.5 (0.7)	1.5 (0.7)	3.0 (1.4)
Belgium (*n* = 1)	4.0	3.0	2.0	4.0	2.0	3.0	1.0
Bulgaria (*n* = 2)	3.0 (1.4)	1.5 (0.7)	1.5 (0.7)	1.0 (0)	1.0 (0)	1.0 (0)	5.0
Croatia (*n* = 19)	2.8 (0.8)	2.8 (1.1)	2.1 (0.8)	2.13 (1.0)	2.3 (0.9)	2.7 (1.0)	4.1 (0.7)
Czech Republic (*n* = 1)	3.0	3.0	1.0	1.0	2.0	4.0	4.0
Denmark (*n* = 1)	4.0	4.0	4.0	4.0	3.0	3.0	4.0
Estonia (*n* = 0)	–	–	–	–	–	–	–
France (*n* = 15)	3.9 (0.9)	2.9 (1.1)	2.6 (1.0)	3.9 (0.6)	2.9 (0.8)	4.0 (1.0)	2.2 (0.8)
Germany (*n* = 4)	3.0 (1.6)	3.5 (1.0)	2.5 (1.0)	3.0 (0.8)	2.3 (0.5)	3.5 (0.6)	2.3 (1.3)
Greece (*n* = 4)	3.0 (1.6)	2.2 (1.5)	2.3 (0.5)	2.8 (1.5)	2.0 (1.4)	3.3 (1.7)	3.3 (1.7)
Hungary (*n* = 4)	3.3 (0.5)	3.0 (0.8)	3.0 (0.8)	2.0 (0)	2.8 (1.0)	2.3 (0.5)	3.8 (0.5)
Ireland (*n* = 1)	2.0	3.0	2.0	4.0	3.0	4.0	2.0
Italy (*n* = 27)	2.5 (1.0)	2.6 (1.0)	2.3 (0.9)	2.5 (1.1)	2.3 (1.0)	3.2 (1.2)	2.8 (1.3)
Lithuania (*n* = 2)	3.0 (0)	4.0 (0)	3.0 (0)	3.0 (0)	2.0 (0)	3.5 (0.7)	3.5 (0.7)
Malta (*n* = 1)	4.0	3.0	2.0	4.0	2.0	4.0	3.0
The Netherlands (*n* = 1)	4.0	5.0	4.0	4.0	4.0	5.0	1.0
Poland (*n* = 0)	–	–	–	–	–	–	–
Portugal (*n* = 28)	3.1 (1.1)	2.7 (1.0)	2.3 (0.9)	3.3 (0.9)	2.2 (0.9)	3.3 (0.9)	3.5 (1.0)
Romania (*n* = 0)	–	–	–	–	–	–	–
Slovenia (*n* = 7)	3.6 (0.9)	2.4 (1.5)	2.2 (1.3)	3.2 (0.8)	2.4 (1.1)	3.8 (0.5)	2.6 (1.3)
Spain (*n* = 8)	3.7 (0.8)	3.3 (0.5)	2.5 (0.5)	2.7 (0.5)	2.7 (0.5)	3.5 (0.8)	21.4 (0.5)
Sweden (*n* = 1)	3.0	2.0	1.0	4.0	1.0	4.0	3.0
United Kingdom (*n* = 1)	3.0	3.0	2.0	4.0	2.0	3.0	1.3 (0.6)
European Economic Area countries and Switzerland							
Switzerland (*n* = 12)	3.6 (0.8)	3.7 (1.0)	3.2 (0.8)	4.3 (0.9)	3.1 (1.1)	4.0 (0.8)	2.2 (1.1)
Norway (*n* = 11)	3.6 (0.8)	3.2 (0.8)	2.6 (0.7)	3.6 (0.9)	2.4 (0.6)	3.6 (0.9)	2.6 (1.1)
Others							
Azerbaijan (*n* = 0)	–	–	–	–	–	–	–
Belarus (*n* = 2)	4.0 (0)	2.5 (0.7)	2.0 (1.4)	3.5 (0.7)	3.0 (0)	3.5 (0.7)	3.5 (0.7)
Bosnia and Herzegovina (*n* = 0)	–	–	–	–	–	–	–
Kosovo (*n* = 1)	4.0	No ID participant	2.0	2.0	4.0	4.0	5.0
Macedonia (*n* = 0)	–	–	–	–	–	–	–
Turkey (*n* = 5)	3.5 (1.0)	3.3 (1.0)	2.5 (0.6)	2.8 (1.5)	3.0 (0.8)	3.0 (0.6)	2.3 (1.0)

Across specializations, low-level implementation of the training curriculum in the real-life setting was identified as the major reason for dissatisfaction by 37.6 % of the respondents, followed by the lack of possibility to do rotation outside the hospital (30.6 %), inadequate supervision by the mentor (30.3 %), and low quality of training curriculum (27.5 %). Sixteen percent of the respondents reported that there is no reason to be dissatisfied.

There was a significant difference between regions regarding satisfaction with the training scheme, with the mean (SD) satisfaction score from high to low being: Western [3.8 (0.7)], Northern [3.8 (0.9)], Eastern [3.4 (0.7)], Other Europe [3.2 (0.9)], and Southern [3.1 (1.1)].

### Training adequacy

Only training on infection control was considered as rather adequate [mean 3.7 (0.7)] by CM residents (Table 2). Among countries with at least five respondents, the highest rate on training in infection control was given by CM trainees from The Netherlands [4.2 (0.4)], whereas the lowest rate was given by residents from Hungary [2.7 (1.2)]. The training in management (administration) [2.9 (0.9)], transplantation medicine [2.6 (1.0)], and health economics [2.6 (1.0)] were considered as rather inadequate by CM residents.

The results from ID respondents showed comparable results, except for transplantation medicine (Table 3). Transplantation medicine was better rated by ID trainees [3.1 (1.2)] in comparison to CM trainees.

### Training methods

The majority of the CM and ID trainees experienced theoretical lectures, which were considered useful, but only a minority of all respondents would like to receive this type of education more (Table 4). Similarly, the majority of CM and ID trainees experienced seminars and rated these 3.9 (0.7) and 4.0 (0.8), respectively. Among all respondents, only a minority would like to experience more seminars.

**Table 4:** Teaching methods experienced by clinical microbiology and infectious disease trainees

	Clinical microbiology trainees			Infectious disease trainees		
	% used this type of training	Rating by respondents who experienced the type of training (SD)	% of respondents who would like to use more of this type of training	% used this type of training	Rating by respondents who experienced the type of training (SD)	% of respondents who would like to use more of this type of training
Theoretical lectures	65.6	3.9 (0.8)	22.8	61.6	3.9 (0.8)	26.0
Seminars	73.1	3.9 (0.7)	22.8	73.3	4.0 (0.8)	36.3
Practical exercises	67.4	4.4 (0.8)	55.4	38.4	4.5 (0.8)	59.4
E-learning activities	18.7	3.7 (0.9)	43.5	17.1	4.0 (0.9)	44.5
Exchange programs abroad	9.8	4.2 (1.1)	65.3	15.7	4.6 (0.9)	62.9 %

Two-thirds of CM residents reported that they had practical exercises during their training, which was in contrast to 38.4 % among ID residents. Both considered this part of the training as useful, 4.4 (0.8) for CM and 4.5 (0.8) for ID respondents. Just half of the respondents wanted to have more practical exercises during their training.

E-learning during the training was experienced by only a minority of the respondents and rated as being useful [3.7 (0.9) for CM and 4.0 (0.9) for ID]. Among all respondents, 43.5 % of CM and 44.5 % of ID trainees want to have more e-learning. Like e-learning, the opportunity to spend time abroad was experienced by a minority of the respondents, and it was considered as useful [4.2 (1.1) by CM and 4.6 (0.9) by ID trainees] by those who have been abroad for their training. The respondents would like to receive more opportunity to spend a part of their training abroad (65.3 % for CM and 62.9 % for ID).

Regarding weekly activities, 40.9 % of CM and 40.3 % of ID respondents reported that they had didactic sessions by an attending physician (staff physician). Didactic sessions by residents were reported by 47.2 % and 41.8 % for CM and ID respondents, respectively. A minority of the respondents experienced weekly journal club meetings, i.e., in 25.6 % of CM respondents and in 26 % of ID respondents. Other activities such as study groups and quizzes were experienced only by less than 13 % and 6 % of the respondents, respectively.

Only a small minority (5.3 %) of CM or ID trainees did not have access to any online journals. More than half of the trainees (59.7 %) mentioned that they regarded information from the internet as a good guidance for their clinical decision in diagnosing and treating patients in most cases.

### Mentorship

In this survey, 61.8 % and 71.5 % of CM and ID respondents mentioned that they had a mentor during their training, respectively. One of five respondents with a mentor could choose their own mentor. The respondents with the mentor reported a mean (SD) satisfaction level of 3.6 (1.1). The frequency of meetings with the mentor was mostly less than once per month (mentioned by 44 % of the respondents with a mentor). Seven percent of CM and 9.7 % of ID residents with a mentor never had a meeting with this person.

More than half of the trainees would like their mentor to be more involved in helping with future career plans (63.5 % of CM and 53.4 % of ID respondents) and in developing practical skills (53.0 % of CM and 61.2 % of ID respondents). A need for more involvement in theoretical knowledge was mentioned by 49.6 % of CM and 54.4 % of ID respondents. One-third of the trainees responded that their mentor should be more involved in the development of communication skills. The majority of the survey participants (89.3 %) agreed fully or partially with their mentor’s periodic evaluations.

### Assessing competency

Theoretical knowledge was assessed during the training in more than 90 % of CM and ID respondents, mostly once a year (38.8 % of CM, and 74.6 % of ID respondents). Practical assessments were experienced by 64.7 % and 51.5 % of the CM and ID trainees, respectively, mostly once a month for CM residents (52.7 %) and once a year for ID residents (78.8 %). Theoretical assessment was considered as useful by the respondents [3.8 (0.8)], whereas practical assessment was considered as less useful [2.8 (1.2)].

To monitor progress, direct observation was the most frequently used method (67.1 % of CM and 43.9 % of ID respondents). Logbook (portfolio) was the second method to monitor progression (in 45.9 % of CM and in 31.8 % of ID respondents). Continuing professional development activities were experienced by 37.6 % of CM and by 40.9 % of ID respondents. 360° evaluation, where the trainee, technologist, and trainer can evaluate each other [5] were used in 14.1 % of CM and in 19.7 % of ID respondents. Such evaluation was used often for evaluating CM training in The Netherlands, where a third of respondents reported this method of progress assessment. The use of the CME system to evaluate progression was experienced by only 6 % of the residents.

About one-fourth of the trainees (27.7 %) had no mandatory final evaluation at the end of the training. Final exams were most often in the format of oral exams, especially for ID respondents (81.1 %). For CM respondents, this number was 52.5 %. A mandatory final written exam was mentioned only by 26.4 % of CM and 29.9 % of ID trainees. Two-thirds of all respondents agreed that a European examination should be developed. Yet, slightly more than half of the respondents who agreed mentioned that such an exam should not be mandatory.

## Discussion

The fields of CM and ID are rapidly evolving and training curricula should keep up with these changes. The opinions of trainees and recently graduated medical specialists are important to identify both strengths as well as opportunities for improvement in the current training curriculum. To the best of our knowledge, this is the first large survey among CM and ID trainees in Europe and several of the findings are worthy of further discussion.

According to this survey, the trainees in Europe tended to be satisfied with their current training program, although they identified particular areas as being inadequate, including management (administration) and health economics. Training in management is important, especially for CM trainees, because they will supervise a microbiology laboratory after finishing the training [5]. Training in health economics can also be combined with training in other areas within the field of CM/ID, for example with antibiotic stewardship/infection control. This training combination has been carried out in Canada [9]. While in general European CM and ID trainees were rather satisfied with their training program, we noticed regional differences in this satisfaction level. Collaboration projects among European countries may contribute to improve training programs and finding aspects of training schemes that can be improved. It is shown that not only training satisfaction varies in Europe, but also training adequacy and training methods.

This survey identified the limited use of modern learning methods such as e-learning in CM/ID training programs, which is in contrast to other medical fields, where distance learning is widespread [10, 11]. However, e-learning is much appreciated by those who have experienced it, and half of them would like to use more of this learning modality. E-learning has several clear benefits, including the possibility to study at one’s own pace and can be followed by residents located at multiple sites. This survey also revealed the lack of scientific activities. Only a quarter of the respondents experienced journal club meetings. Since evidence-based medicine is continuously evolving and requires regular updates, journal clubs may contribute to the education of trainees in their abilities to adequately assess new information derived from new studies. The trainees value the possibility to go abroad as part of their training, which is understandable, since infectious diseases do not recognize geographical borders. Financing and implementing such a cross-border training in the training curriculum requires further discussions among policymakers.

This survey showed that the assessments of the trainees are performed mainly using classical methods such as direct observation. Logbooks are widely used but CME has not found its way into CM/ID. In our survey, the majority of the trainees does agree with the idea of having a European final examination, as in ophthalmology and vascular surgery [12, 13]. Examination in these medical fields is not compulsory but offers recognition of competence and facilitates movement between European countries. The added value of a European examination was shared by CM and ID trainees who participated in the survey.

Like in many other medical specializations [14–17], it is common to assign a mentor to a CM and ID trainee. The successful mentoring program has been linked to several beneficial outcomes for trainees, such as improved career satisfaction [14]. Trainees need guidance from their mentor on future career plans and on technical aspects of the field. To a lesser extent, a minority of the trainees would like to receive help from their mentor regarding communication aspects. Communication skills are important for performing the tasks of CM or ID specialists [5]. The need for a mentor in CM/ID training has been recognized by the ESCMID, which recently launched a mentorship program that has approved 20 mentorship centers across Europe [18].

In the present survey, up to one-third of the trainees did not know about the accreditation status of their training program. In fact, not all residency programs are accredited. Accreditation of a training program is needed in order to improve quality and facilitate exchange of trainees between institutions [5, 19].

This survey has an important limitation that needs to be acknowledged. The participants were self-selected and the study sample did not include participants without internet access. These aspects may have led to a lack of representativeness of the sample and over-/under-representation of some countries. Yet, we believe that our active approach, where local TAE representatives in almost all countries in Europe were involved, has reached a large part of the trainees. Unfortunately, no data on the overall number of trainees in every country in Europe are available, so we cannot estimate the response rate of this survey. Also noteworthy of mention is that not all CM participants were medical doctors; in several countries such as Belgium and France, a pharmacist can be also a trainee in CM.

To the best of our knowledge, no comparable extensive survey in other medical specialties in Europe has been performed. The present survey may inspire similar studies among other clinical specialties, allowing future comparisons between specialties. Comparison between specialties regarding satisfaction of the training curriculum might help junior doctors’ decisions to pursue certain specialties because satisfaction among current trainees can be an important factor for such a decision. In the USA, several surveys among junior doctors, residents, and young trainees have been performed recently to attract more junior doctors to choose CM and ID as a their medical specialty [20–22].

In conclusion, this survey shows heterogeneity in training conditions in European countries, identifies perceived gaps in training, and suggests gray areas worthy of improvements. In an era of cumulative global efforts to reduce morbidity and mortality related to infections due to multidrug microorganisms and emerging infectious diseases, improvements of training in ID and CM should be a key component of a multifaceted European program.

## Electronic supplementary material

Below is the link to the electronic supplementary material.

ESM 1 (DOCX 150 kb)
