# Supplementary material for: A large survey among European trainees in clinical microbiology and infectious disease on training systems and training adequacy: identifying the gaps and suggesting improvements
Source: Eur J Clin Microbiol Infect Dis. 2016 Oct 4;36(2):233–42. doi: 10.1007/s10096-016-2791-9 (PMC5253151; doi:10.1007/s10096-016-2791-9)
Supplement: Supplementary file 1 — (DOCX 150 kb) [file 10096_2016_2791_MOESM1_ESM.docx]

| About you |
| --- |

**Country where you work?**

**Age?**

**Gender?**

**Where do you work?**

Please choose **only one** of the following:

- University / large county hospital
- Non university hospital with less than 750 beds
- Non university hospital with more than 750 beds
- Private clinic

**Speciality:**

Please choose **only one** of the following:

- CM trainee
- Young CM specialist < 3 years of specialization
- ID trainee
- Young ID specialist < 3 years of specialization
- CM/ID trainee / young specialist < 3 years of specialization
- Other

**What is your average monthly income - gross salary (please denote the currency)?**

Please write your answer here:

**How long was the period between your graduation from medical school and the start of your traineeship?**

Please write your answer here:

**Is your training programme accredited?**

Please choose **only one** of the following:

- Yes
- No
- I don’t know

| Training satisfaction |
| --- |

**How satisfied are you with your training / residency scheme?**

Please choose **only one** of the following:

- Completely dissatisfied
- Dissatisfied
- Neither satisfied nor dissatisfied
- Satisfied
- Completely satisfied

**What is the major cause of dissatisfaction with your training programme?**

Please choose **all** that apply:

- Low quality of official training curriculum
- Low level of implementation of curriculum in real-life setting
- Inadequate supervision by your mentor
- No education/rotation outside of your hospital
- There is nothing that causes dissatisfaction
- Other:

| Training adequacy |
| --- |

**Please indicate how adequate your training is regarding:**

Please choose the appropriate response for each item:

|  | **Completely inadequate** | **Inadequate** | **Neither inadequate nor adequate** | **Adequate** | **Completely adequate** |
| --- | --- | --- | --- | --- | --- |
| **Information systems** |  |  |  |  |  |
| **Practice economics** |  |  |  |  |  |
| **Travel medicine** |  |  |  |  |  |
| **Administration** |  |  |  |  |  |
| **Infection control** |  |  |  |  |  |
| **Transplantation/other immunosuppressed patients** |  |  |  |  |  |

| Training methods |
| --- |

**What type of teaching methods are used during your training?**

Please choose **all** that apply:

- Theoretical lectures
- Seminars
- Practical exercises
- E-learning activities
- Exchange programmes abroad

**What type of teaching methods would you like to see used more during your training?**

Please choose **all** that apply:

- Theoretical lectures
- Seminars
- Practical exercises
- E-learning activities
- Exchange programmes abroad

**Please indicate how useful you find the following teaching methods during your training:**

Only answer this question for the items you selected in the previous question.

|  | **Completely useless** | **Useless** | **Neither useful nor useless** | **Useful** | **Completely useful** |
| --- | --- | --- | --- | --- | --- |
| **Theoretical lectures** |  |  |  |  |  |
| **Seminars** |  |  |  |  |  |
| **Practical exercises** |  |  |  |  |  |
| **E-learning activities** |  |  |  |  |  |
| **Exchange programmes abroad** |  |  |  |  |  |

**What type(s) of weekly education activities does your programme provide for residents and fellows:**

Please choose **all** that apply:

- Weekly didactic sessions (given by the attending)
- Weekly didactic sessions (given by residents)
- Weekly quizzes
- Study groups
- Conventional journal club
- Web-based journal club
- Other:

**In the past month, did you use the Internet as a source of information for your clinical work?**

Please choose **only one** of the following:

- Yes
- No

**Do you consider information from the Internet a good source of guidance for your clinical decisions?**

Please choose **only one** of the following:

- Never
- Sometimes
- About half the time
- Most of the time
- Always

**Do you have access to relevant and recent literature covering your specialty during training?**

Please choose the appropriate response for each item:

|  | **not at all** | **some literature** | **most literature** | **all the literature I need** |
| --- | --- | --- | --- | --- |
| **Textbooks** |  |  |  |  |
| **Journal papers** |  |  |  |  |

| Mentorship |
| --- |

**Do you have a specifically assigned mentor who supervises your progress during your training programme?**

Please choose **only one** of the following:

- Yes
- No
- I don’t know

**Did you have a say in the choice of mentor assigned to you?**

Answer this question only when your answerwas 'Yes' to the question' Do you have a specifically assigned mentor who supervises your progress during your training programme?’

Please choose **only one** of the following:

- yes
- no
- didn't think it was important
- don't know

**How satisfied are you with your mentor?**

Answer this question only when your answerwas 'Yes' to the question' Do you have a specifically assigned mentor who supervises your progress during your training programme?’

Please choose **only one** of the following:

- Completely dissatisfied
- Dissatisfied
- Neither satisfied nor dissatisfied
- Satisfied
- Completely satisfied

**How often do you discuss with your mentor your personal progress and issues that bother you?**

Answer this question only when your answerwas 'Yes' to the question' Do you have a specifically assigned mentor who supervises your progress during your training programme?’

Please choose **only one** of the following:

- Every day
- Once or twice per week
- Several times per month
- Once per month
- Less than once per month
- Never

**Which aspect of the training should your mentor be more involved in?**

Answer this question only when your answerwas 'Yes' to the question' Do you have a specifically assigned mentor who supervises your progress during your training programme?’

Please choose **all** that apply:

- Theoretical knowledge
- Practical skills
- Communication skills
- Help with further career plans
- Other:

**Your mentor's contribution to your progress during the training is:**

Answer this question only when your answerwas 'Yes' to the question' Do you have a specifically assigned mentor who supervises your progress during your training programme?’

Please choose **only one** of the following:

- Completely insignificant
- Insignificant
- Neither insignificant nor significant
- Significant
- Completely significant

**Do you agree with the periodic evaluations given by your mentor?**

Answer this question only when your answerwas 'Yes' to the question' Do you have a specifically assigned mentor who supervises your progress during your training programme?’

Please choose **only one** of the following:

- yes
- partially
- no
- I don't know

| Assessing competency |
| --- |

**Do you have mandatory in-training assessments?**

Please choose **only one** of the following:

- Yes
- No

**Which aspects of your progress are assessed during you training?**

Please choose **all** that apply:

- Theoretical knowledge
- Practical skills

**How often do you have:**

Answer this question when you had 'Practical skills' or 'Theoretical knowledge'

|  | **Never** | **Once a year** | **Once every few months** | **Once a month** | **Several times a month** | **Once a week** |
| --- | --- | --- | --- | --- | --- | --- |
| **...theoretical assessments during your training?** |  |  |  |  |  |  |
| **...practical assessments during your training?** |  |  |  |  |  |  |

**Do you find:**

|  | **Completely useless** | **Useless** | **Neither useful nor useless** | **Useful** | **Completely useful** |
| --- | --- | --- | --- | --- | --- |
| **...theoretical assessments useful for your progress during training?** |  |  |  |  |  |
| **...practical assessments useful for your progress during training?** |  |  |  |  |  |

**Which methods are used for assessment of your progress?**

Please choose **all** that apply:

- e-logbook/portfolio
- quizzes with multiple choice questions
- CME products
- directly observed procedures
- continuing professional development activities
- 360 ˚appraisal

**Is there a mandatory written exam at the end of your training?**

Please choose **only one** of the following:

- Yes
- No

**Is there a mandatory oral exam at the end of your training?**

Please choose **only one** of the following:

- Yes
- No

**Do you agree with the idea of a common European exam?**

Please choose **only one** of the following:

- Yes, it should be mandatory
- Yes, but it should not be mandatory
- No, I don't agree
- I don't know/haven't decided yet
